# The effect of aging on hardness of heat cured denture base resin modified with recycled acrylic resin

**DOI:** 10.1002/cre2.828

**Published:** 2024-02-06

**Authors:** Amrah Y. AL‐Jmmal, Nada Z. Mohammed, Hala M. AL‐kateb

**Affiliations:** ^1^ Department of Prosthetic Dentistry University of Mosul Mosul Iraq

**Keywords:** hardness, heat cured resin, recycled acrylic resin

## Abstract

**Background:**

The second rule of the 4Rs concept (Reduce, Reuse, Recycle, and Recover) was applied in this study using recycled acrylic resin to improve the hardness and study the effect of aging on the hardness of heat cured denture base resins.

**Method:**

Forty heat‐cured acrylic resin samples were prepared and divided into control and modified groups. The hardness was tested using a type D durometer hardness tester for evaluating the effect of the thermal aging process on the hardness in the control and modified groups. The samples were either subjected to thermal aging (the specimens thermo‐cycled 10 cycles per day between 55°C and 5°C with a 30‐s dwell time) or were not.

**Results:**

The mean difference in hardness between specimens with and without aging in the modified group increased with increasing concentrations of incorporated recycled acrylic resin. Independent samples *t* test revealed that the hardness values of modified groups with aging were significantly higher than in those without aging (*p* ≤ 0.05). ANOVA revealed that the modified group revealed a significant increase in hardness than that of the control group (*p* ≤ 0.05).

**Conclusions:**

Recycling and reuse of acrylic resins improved the hardness of denture base resins. The aging period significantly affected the hardness values of the control and modified groups.

## 1 INTRODUCTION

Environmental concerns regarding pollution have become a serious concern over the last decade (Aseel & Saja, 2020). Effective management of waste materials by recycling, in an attempt to reduce environmental pollution, reduces the damage to human health and exhaustion of natural resources (Agarwal et al., 2019).

Heat cured denture base resins are biocompatible materials that are widely used for the construction of denture bases (Naji, 2020). Because these types of denture base materials do not satisfy all intended mechanical properties, these are reinforced by the incorporation of fibers, fillers, metal wires or plates, and nano‐scaled reinforcing materials to improve their physicomechanical properties (Djustiana et al., 2020; Gungor et al., 2017; Mohammed & Hasan, 2022a; Raszewski, 2020; Shahabi et al., 2021; Tokar et al., 2018). The denture base material in the oral cavity undergoes a change in its thermal properties during eating or intake of different types of fluids, which may affect the hardness of the resin material. Therefore, predicting the behavior of denture materials is challenging (Wang et al., 2014).

*Aim*: The second rule of the 4Rs concept (Reduce, Reuse, Recycle, and Recover) was applied in this study by using recycled acrylic resin to improve the hardness and to study the cumulative effect of aging resulting from fluctuating oral temperature due to the intake of cold and hot food and fluids on the hardness of the acrylic resin denture base.

## 2 MATERIALS AND METHODS

### 2.1 Study groups

Forty specimens were prepared from a heat‐cured acrylic resin (SpofaDental) by creating a stone mold in a metallic dental flask using a plastic master mold. The specimens were grouped as follows:

a. Control group.
b. Modified group: incorporated with recycled acrylic resin (Chaini‐HKG) at 1%, 3%, and 5% by volume.

### 2.2 Sample manufacturing

**Figure 1:**
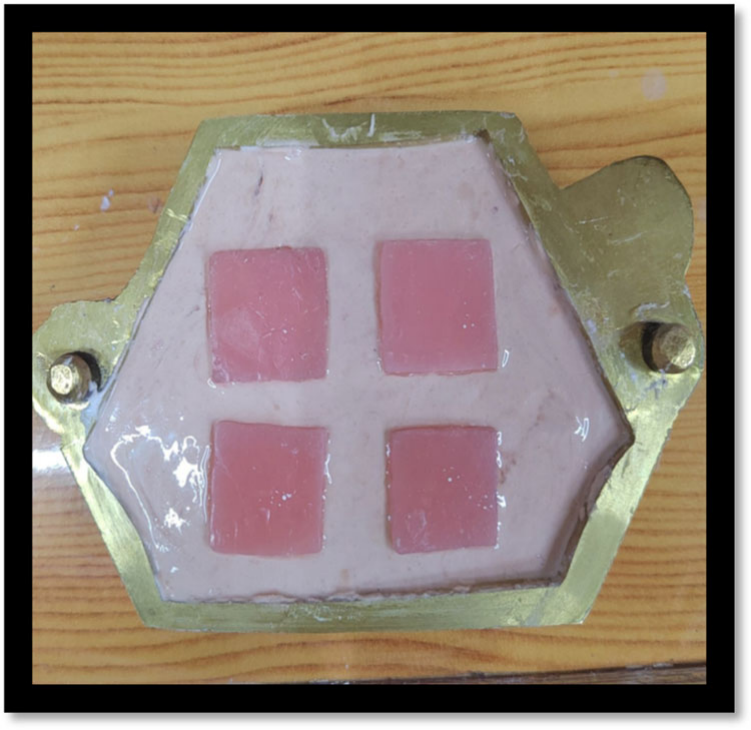
Master molds in dental flask.

The master models were prepared with dimensions of 10 (length) × 10 (width) × 3.3 (height) ±0.2 mm (Song et al., 2019) and embedded into the lower half of the dental flask after being filled with freshly mixed die‐stone (Figure 1) according to the manufacturer's instructions (water powder ratio of 100:23). After complete setting, the diestone surface was painted using an alginic isolator. The upper compartment of the dental flask was then completely filled with the die stone. After the complete setting of the die stone, the master molds were carefully removed, and the dental flask was packed with acrylic resin dough (Stewart & Bagby, 2013).

To prepare the control group specimens, the powder–liquid mixing ratio recommended by the manufacturer was followed. The specimens of the modified group were prepared by replacing a volume percent of the methyl methacrylate (PMMA) liquid with an equal volume percent of recycled acrylic resin (Hayran & Keskin, 2019). First, measured amounts of heat‐cured monomer and recycled acrylic resin were mixed until a homogenous mixture was obtained. Subsequently, the heat‐cured acrylic resin powder was gradually added. When the dough stage was reached, it was packed into the prepared dental flask, which was closed under a pressure of 200 MPa and processed in a water bath using a short curing cycle (Van Noort, 2013).

### 2.3 Hardness

**Figure 2:**
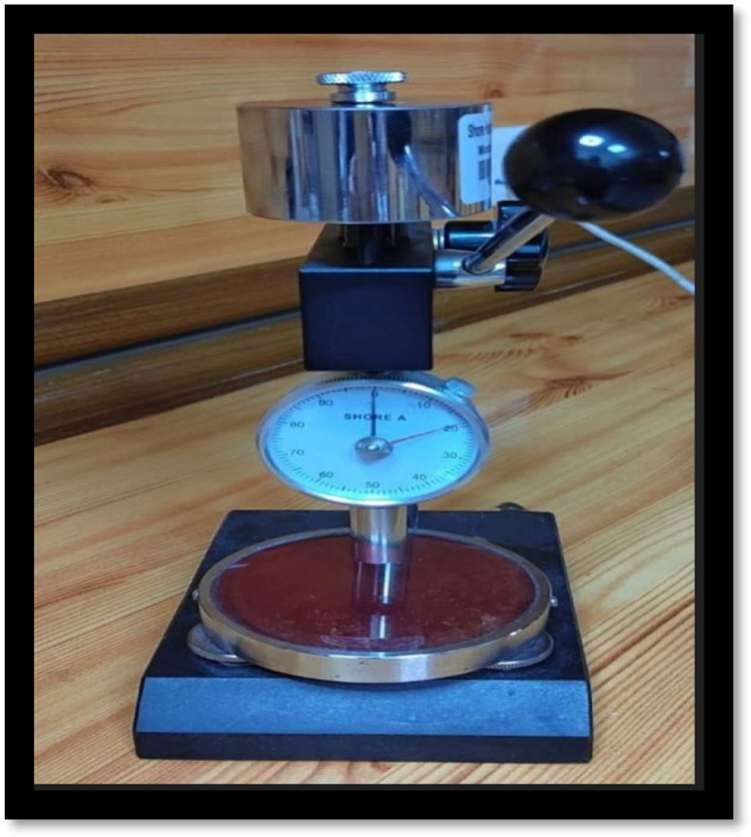
Type D durometer hardness tester.

The surface hardness of the specimens was measured using a type D durometer hardness tester (Salman et al., 2017) equipped with a 1.25 mm round steel ball indenter (Figure 2). The needle of the tester was held 12 mm away from the surface of the tested specimen. The mean of five measurements from different areas on the surfaces of the tested specimens was recorded (Song et al., 2019).

### 2.4 Thermal cycles

For evaluating the effect of the aging process on the hardness values in the control and modified groups, the specimens were either not aged (immersed in distilled water only) or aged by thermal immersion (the specimens thermo‐cycled 10 cycles per day between 55°C and 5°C with a 30‐s dwell time) for one month (300 cycles), three months (900 cycles), six months (1800 cycles), one year (3650 cycles), and two years (7300 cycles) (Rahaman Ali et al., 2020).

### 2.5 Statistical analysis

The obtained data were statistically analyzed using SPSS 19.0 software (IBM Corp.) using descriptive statistics to determine the effect of incorporating recycled acrylic resin (at 1%, 3%, and 5%) on the hardness of the heat cured denture base resin.

An independent sample *t* test was performed to determine whether there was a statistically significant change in the hardness of the control and modified groups after thermal aging at a significance level of *p* ≤ 0.05. ANOVA and Duncan test were applied to assess whether the thermal aging process significantly affected the hardness of the control and modified groups at different aging periods (one month, three months, six months, one year, and two years (*p* ≤ 0.05).

## 3 RESULTS

The mean hardness values of the control and modified groups with and without thermal aging (Table 1) revealed that the hardness of the heat cured denture base resin increased with the incorporation of recycled acrylic resin. The increase in the hardness of the modified group was directly related to the concentration of the incorporated recycled acrylic resin. The hardness of the modified group with thermal aging was higher than that of the group without thermal aging for all aging periods (one month, three months, six months, one year, and two years).

**Table 1:** Mean ± SD and independent samples *t* test for the hardness of control and modified groups without and with thermal aging after all aging period.

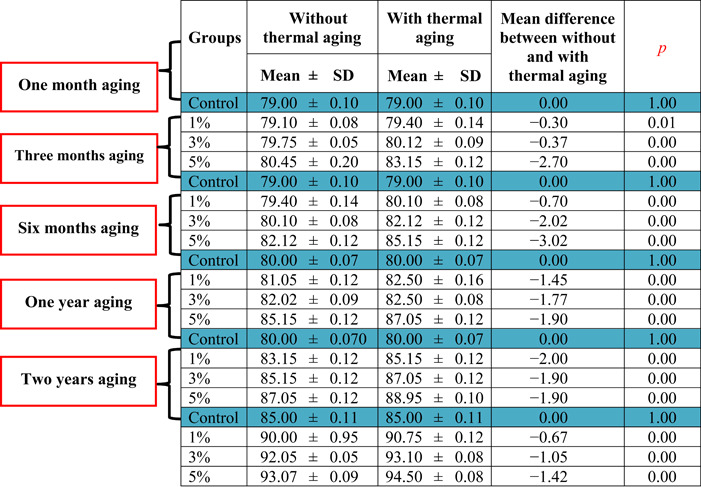

Abbreviation: SD, standard deviation.

The mean difference between the hardness of the thermally aged and nonthermally aged specimens in the modified group increased with increasing concentration of the incorporated recycled acrylic resin. Independent sample *t* tests (Table 1) demonstrated statistically significant differences in the hardness of modified groups with thermal aging than in those without thermal aging. The hardness values of the control group were not affected by thermal aging within the specific aging period (*p* ≤ 0.05).

**Figure 3:**
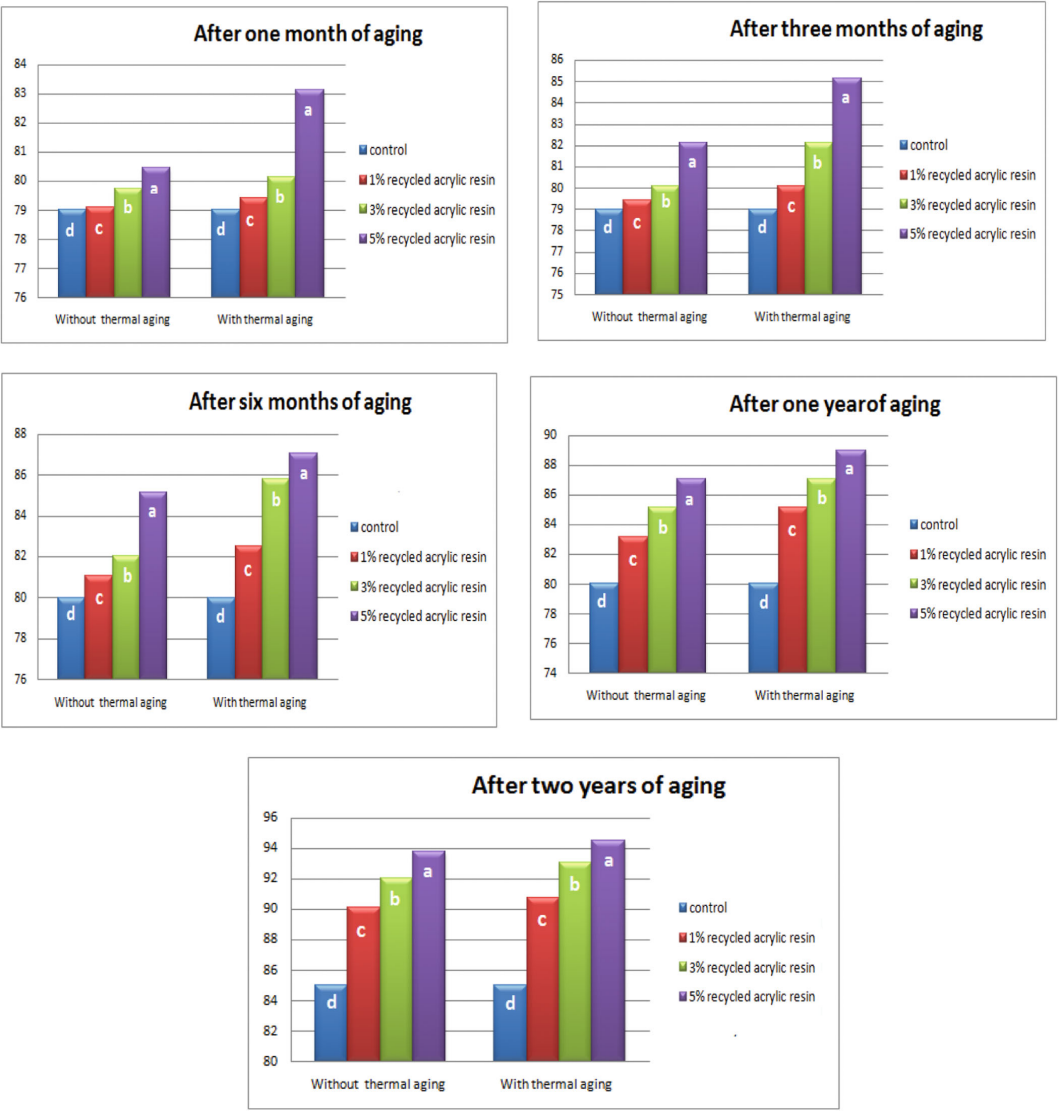
Duncan's test for the hardness of the control and modified groups with and without thermal aging.

ANOVA (Table 2) and Duncan test (Figure 3) showed that the hardness was significantly higher in the modified group than in the control and aging groups (*p* ≤ 0.05).

**Table 2:** ANOVA for the hardness of control and modified groups without and with thermal aging.

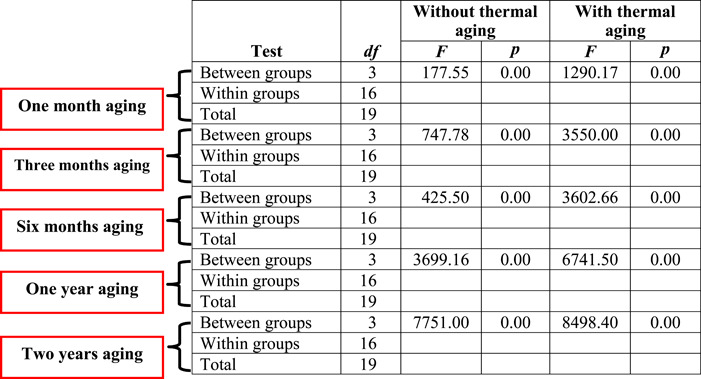

Abbreviation: *df*, degrees of freedom.

**Figure 4:**
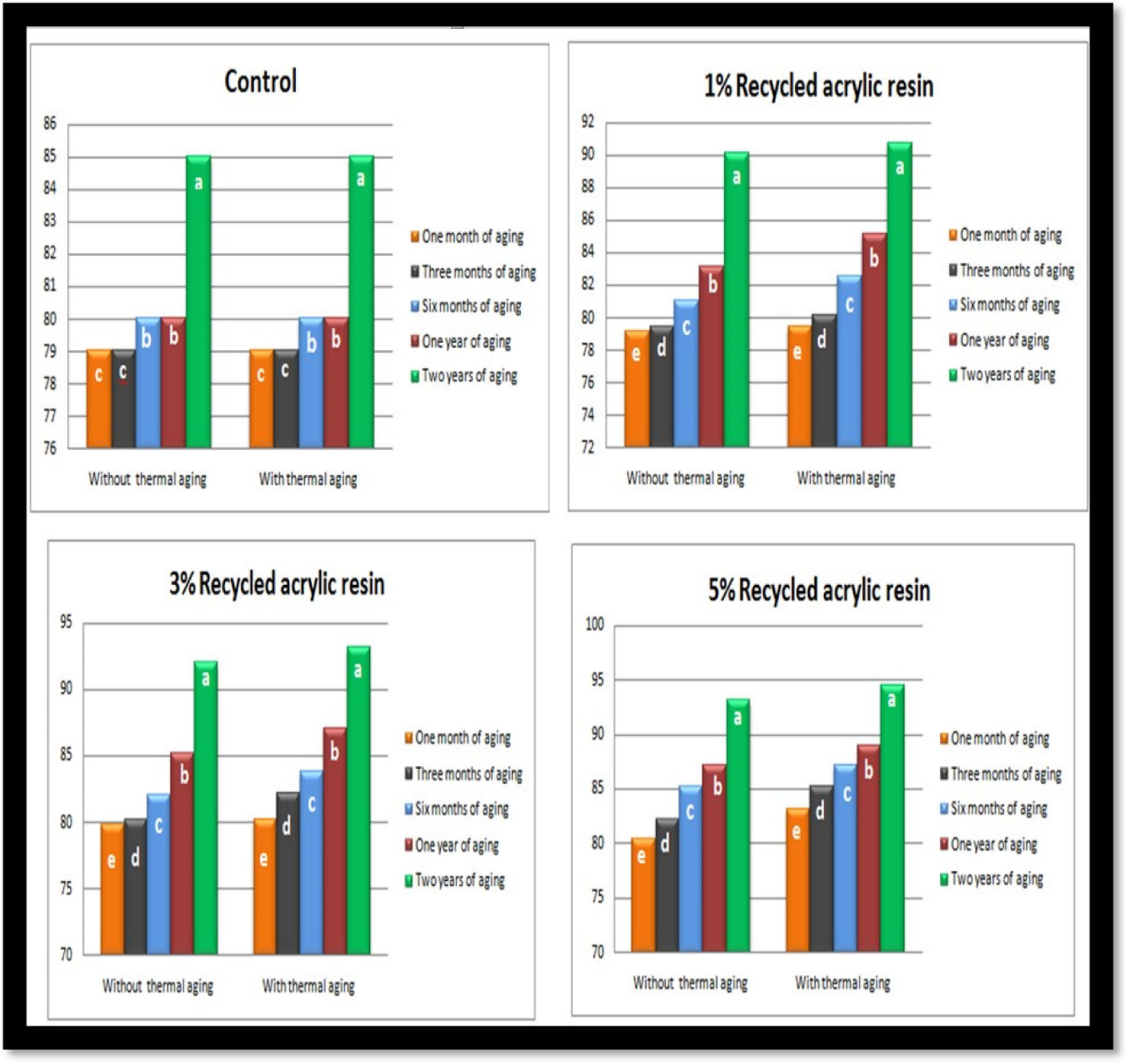
Duncan's test for the effect of the aging period on the hardness of the control and modified groups.

The results of this study show that the hardness of the control and modified groups increased with the aging period. ANOVA (Table 3) and Duncan test (Figure 4) revealed that the hardness of the control and modified groups was significantly affected by the aging periods (*p* ≤ 0.05).

**Table 3:** ANOVA for the effect of aging periods on the hardness of control and modified groups.

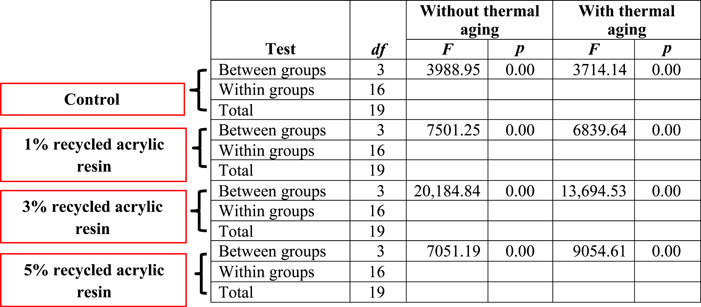

Abbreviation: *df*, degrees of freedom.

## 4 DISCUSSION

Recycling and reusing dental materials have substantial economic and environmental advantages (Nandish et al., 2013). Simulating the complex oral environment in vitro is a major limitation of our study. However, in this study, the thermal aging process was attempted to match the fluctuations in the mouth temperature.

The incorporation of the recycled acrylic resin into the heat cured denture base resin improved its hardness (Table 1), which is consistent with the results obtained by Salim and Muhsin (2020), who concluded that the incorporation of a recycled polyether ether ketone polymer improved the hardness of the denture resin. The enhanced hardness could be attributed to the improved degree of conversion, high crystallinity, and/or decreased porosity of the resulting composite polymer (Dagdiya et al., 2019; Elboraey et al., 2016; Mohammed & Hasan, 2022b), or it might be due to the decrease in the residual monomer content (Ajay et al., 2020). The highest hardness was recorded by adding 5% recycled acrylic resin. Increasing the concentration of homogenously diffused fillers within the polymeric resin matrix increases the hardness of the polymeric material (Alhareb et al., 2018).

Table 1 shows that the hardness of the modified group with thermal aging was higher than that without thermal aging for all aging periods, possibly due to an improved degree of conversion secondary to complementary polymerization (Goiato et al., 2010). This result differed from that obtained by Atalay et al. (2021), who used the Knoop microhardness test under a 25 g load to evaluate the hardness of acrylic resin after 5000 cycles and observed a decrease in the hardness of acrylic resin samples after thermal cycling; few were not statistically significant. Çakmak et al. (2023) found that thermal aging significantly decreased the hardness of grapheme‐reinforced polymethyl methacrylate, whereas its effect on the hardness of 3D‐printed acrylic resin was not statistically significant.

The mean difference between the hardness of the thermally aged modified group and that of the group without aging (Table 1) increased with increasing concentrations of incorporated recycled acrylic resin. The hardness of the control group was not affected by thermal aging. The improved structural integrity of the modified group resulted from the incorporation of the recycled acrylic resin, which impeded the leaching of unreacted monomers that would be consumed in the complementary polymerization initiated by thermal aging (Gungor et al., 2017).

Independent sample t‐tests showed that the hardness values were significantly higher in the thermally aged modified group than those without (*p* ≤ 0.05) (Table 1). This is consistent with the results obtained by Melo Neto et al. (2023), who reported a statistically significant increase in the hardness of polyethylene terephthalate glycol with polyurethane and acrylic resin after thermal cycling. The growing polymeric chain resulting from complementary polymerization entangled with the original resin matrix in a manner that increased their physical crosslinking (Hamouda, 2018).

ANOVA (Table 2) and Duncan test (Figure 3) revealed a significant increase in the hardness of the modified group with increasing concentration of incorporated recycled resin as compared to that of control for both age groups (*p* ≤ 0.05). The resistance of the resin matrix to deformation may be the cause of the extra energy needed by polymers with interpenetrating networks to break such bonds (Oleiwi et al., 2018). This result agrees with that of Al‐Jmmal et al. (2020), who found that as recycled PMMA concentration increased, acrylic resin hardness increased significantly. This, however, is contrary to the findings of Gad et al. (2018), who discovered that adding thymoquinone significantly decreased the hardness of acrylic resin specimens. Because of the fillers' forced entry into the polymeric resin chains, there is a decrease in entanglement and secondary bonding between the chains (Chladek et al., 2016; Mosalman et al., 2017).

The results of the research displayed that as the aging period increased, correspondingly increased the hardness of the control and modified groups. According to Mohammed's (2013) research, immersion times have an important effect, especially when paired with thermal stress. The hardness of the control and modified groups is significantly (*p* ≤ 0.05) influenced by the aging periods, as indicated by an ANOVA (Table 3) and Duncan test (Figure 4). Shah et al. (2014) believe that this increase in hardness may be caused by plasticizers leaching out of the polymer matrix. The modified group's lower water sorption as a result of the enhanced physical crosslinking caused by the addition of recycled acrylic resin could be responsible for the modified group's higher hardness overall aging periods compared to the control groups (Das & Barhate, 2019).

## 5 CONCLUSION

The heat cured denture base acrylic resin's hardness has been improved through recycling and reusing acrylic resin. Repeated exposure to heat alteration has an effect on the hardness of the heat cured denture base acrylic resin. The control and modified groups' hardness values were considerably influenced by the aging period.

## AUTHOR CONTRIBUTIONS

Not applicable.

## CONFLICT OF INTEREST STATEMENT

The authors declare no conflict of interest.

## Supporting information

Supporting information.

## Data Availability

Data that support the finding of this study are openly available on request from the corresponding author at: amra2012@uomosul.edu.iq.
